# Ethyl 4-[3-(1*H*-imidazol-1-yl)propyl­amino]-3-nitro­benzoate

**DOI:** 10.1107/S1600536811036300

**Published:** 2011-09-14

**Authors:** Yeong Keng Yoon, Mohamed Ashraf Ali, Tan Soo Choon, Wan-Sin Loh, Hoong-Kun Fun

**Affiliations:** a Institute for Research in Molecular Medicine, Universiti Sains Malaysia, 11800 USM, Penang, Malaysia; b X-ray Crystallography Unit, School of Physics, Universiti Sains Malaysia, 11800 USM, Penang, Malaysia

## Abstract

In the title compound, C_15_H_18_N_4_O_4_, the 1*H*-imidazole ring forms a dihedral angle of 67.12 (8)° with the benzene ring. An *S*(6) ring motif is formed *via* an intra­molecular N—H⋯O hydrogen bond. In the crystal, neighbouring mol­ecules are linked by a pair of inter­molecular N—H⋯N hydrogen bonds, forming an inversion dimer. The dimers are further linked by a pair of C—H⋯O hydrogen bonds, leading to the formation of chain along [021]. A C—H⋯π inter­action involving the centroid of the benzene ring is also observed between the chains.

## Related literature

For applications of phenyl­enediamines, see: Sabelle (2006 ▶); Glebowska *et al.* (2009 ▶); Remusat *et al.* (2004 ▶). For hydrogen-bond motifs, see: Bernstein *et al.* (1995 ▶).
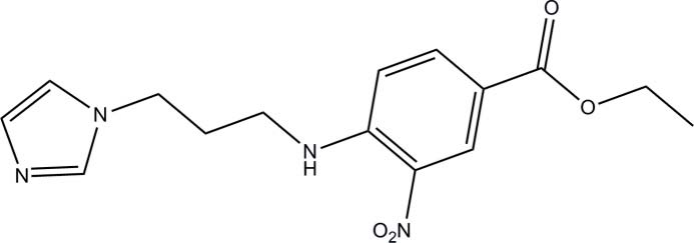


## Experimental

### 

#### Crystal data

C_15_H_18_N_4_O_4_
*M* *_r_* = 318.33
Triclinic, 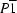

*a* = 8.4860 (4) Å
*b* = 8.6175 (4) Å
*c* = 11.7507 (6) Å
α = 77.489 (1)°
β = 81.732 (1)°
γ = 67.977 (1)°
*V* = 775.83 (7) Å^3^
*Z* = 2
Mo *K*α radiation
μ = 0.10 mm^−1^
*T* = 297 K
0.43 × 0.37 × 0.23 mm

#### Data collection

Bruker SMART APEXII DUO CCD area-detector diffractometer
Absorption correction: multi-scan (*SADABS*; Bruker, 2009 ▶) *T*_min_ = 0.958, *T*_max_ = 0.978
15358 measured reflections
4470 independent reflections
3627 reflections with *I* > 2σ(*I*)
*R*_int_ = 0.021

#### Refinement

*R*[*F* ^2^ > 2σ(*F* ^2^)] = 0.049
*wR*(*F* ^2^) = 0.173
*S* = 1.05
4470 reflections
213 parameters
H atoms treated by a mixture of independent and constrained refinement
Δρ_max_ = 0.31 e Å^−3^
Δρ_min_ = −0.23 e Å^−3^

### 

Data collection: *APEX2* (Bruker, 2009 ▶); cell refinement: *SAINT* (Bruker, 2009 ▶); data reduction: *SAINT*; program(s) used to solve structure: *SHELXTL* (Sheldrick, 2008 ▶); program(s) used to refine structure: *SHELXTL*; molecular graphics: *SHELXTL*; software used to prepare material for publication: *SHELXTL* and *PLATON* (Spek, 2009 ▶).

## Supplementary Material

Crystal structure: contains datablock(s) global, I. DOI: 10.1107/S1600536811036300/is2771sup1.cif

Structure factors: contains datablock(s) I. DOI: 10.1107/S1600536811036300/is2771Isup2.hkl

Supplementary material file. DOI: 10.1107/S1600536811036300/is2771Isup3.cml

Additional supplementary materials:  crystallographic information; 3D view; checkCIF report

## Figures and Tables

**Table 1:** Hydrogen-bond geometry (Å, °) *Cg*1 is the centroid of the C7–C12 benzene ring.

*D*—H⋯*A*	*D*—H	H⋯*A*	*D*⋯*A*	*D*—H⋯*A*
N1—H1*N*1⋯O2	0.859 (18)	2.004 (18)	2.6464 (18)	130.9 (15)
N1—H1*N*1⋯N3^i^	0.859 (18)	2.345 (17)	3.0281 (18)	136.7 (15)
C15—H15*A*⋯O1^ii^	0.96	2.47	3.346 (2)	151
C1—H1*A*⋯*Cg*1^iii^	0.93	2.90	3.5962 (16)	132

Symmetry codes: (i) 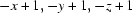; (ii) 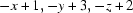; (iii) 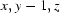.

**Fig. 1.**
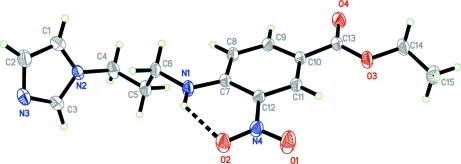
The molecular structure of the title compound, showing 30% probability displacement ellipsoids. The dashed line indicates the intramolecular hydrogen bond.

**Fig. 2.**
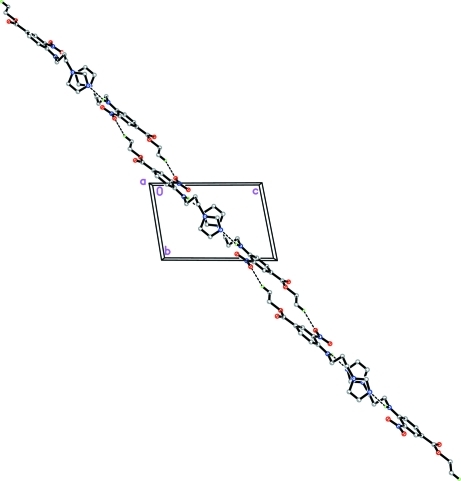
The crystal packing of the title compound, viewed along the a axis. H atoms not involved in the intermolecular interactions (dashed lines) have been omitted for clarity.

## supplementary crystallographic information

### Comment

Nitrophenyleneamine is an important class of compounds in organic synthetic chemistry. They are most of the time used to synthesize phenylenediamines by reducing the nitro (NO_2_) group to amine (NH_2_). Phenylenediamines themselves are then used as composition in making dyes (Sabelle, 2006), metallomesogens (Glebowska *et al.*, 2009) as well as ligand precursors. Condensation of substituted *o*-phenylenediamine with various diketones is then used in the preparation of a variety of pharmaceuticals (Remusat *et al.*, 2004).

In the title compound (Fig. 1), the 1*H*-imidazole (C1/C2/N3/C3/N2) is almost planar with a maximum deviation of 0.003 (2) Å at atom C3 and it forms a dihedral angle of 67.12 (8)° with the benzene ring (C7–C12). An *S*(6) ring motif (Bernstein *et al.*, 1995) is formed *via* an intramolecular N1—H1N1···O2 hydrogen bond (Table 1).

In the crystal packing (Fig. 2), pairs of intermolecular N1—H1N1···N3 and C15—H15A···O1 hydrogen bonds (Table 1) link the neighbouring molecules to form dimers, leading to the formation of chains along the [021]. The crystal packing is further stabilized by a C—H···π interaction (Table 1), involving the centroid of the benzene ring (*Cg*1).

### Experimental

Ethyl-4-fluro-3-nitro benzoate (4.6 mmol) in dichloromethane (20 mL) was added into the solution of 3-(1*H*-imidazole-1yl)propane-1-amine (7.0 mmol) and *N*, *N*-diisopropylethylamine (5.6 mmol) in dichloromethane (20 mL). The reaction mixture was stirred overnight at room temperature. After completion of the reaction, evidenced by TLC analysis. The reaction mixture was washed with water (10 mL × 2) and 10% Na_2_CO_3_ (10 ml × 2). The dichloromethane layer was collected and dried over Na_2_SO_4_. The organic layer was concentrated under reduced pressure to afford white-colored crystals.

### Refinement

Atom H1N1 was located in a difference Fourier map and was refined freely. The remaining H atoms were positioned geometrically and refined using a riding model, with *U*_iso_(H) = 1.2 or 1.5*U*_eq_(C) (C—H = 0.93–0.97 Å). A rotating group model was applied to the methyl group. Three outliners were omitted for the final refinement, 0 -1 4, -5 0 4 and -4 0 5.

### Crystal data

**Table:**

C_15_H_18_N_4_O_4_	*Z* = 2
*M**_r_* = 318.33	*F*(000) = 336
Triclinic, *P*1	*D*_x_ = 1.363 Mg m^−^^3^
Hall symbol: -P 1	Mo *K*α radiation, λ = 0.71073 Å
*a* = 8.4860 (4) Å	Cell parameters from 6626 reflections
*b* = 8.6175 (4) Å	θ = 2.6–32.5°
*c* = 11.7507 (6) Å	µ = 0.10 mm^−^^1^
α = 77.489 (1)°	*T* = 297 K
β = 81.732 (1)°	Block, yellow
γ = 67.977 (1)°	0.43 × 0.37 × 0.23 mm
*V* = 775.83 (7) Å^3^	

### Data collection

**Table:**

Bruker SMART APEXII DUO CCD area-detector diffractometer	4470 independent reflections
Radiation source: fine-focus sealed tube	3627 reflections with *I* > 2σ(*I*)
graphite	*R*_int_ = 0.021
φ and ω scans	θ_max_ = 30.0°, θ_min_ = 2.6°
Absorption correction: multi-scan (*SADABS*; Bruker, 2009)	*h* = −11→11
*T*_min_ = 0.958, *T*_max_ = 0.978	*k* = −11→12
15358 measured reflections	*l* = −16→16

### Refinement

**Table:**

Refinement on *F*^2^	Primary atom site location: structure-invariant direct methods
Least-squares matrix: full	Secondary atom site location: difference Fourier map
*R*[*F*^2^ > 2σ(*F*^2^)] = 0.049	Hydrogen site location: inferred from neighbouring sites
*wR*(*F*^2^) = 0.173	H atoms treated by a mixture of independent and constrained refinement
*S* = 1.05	*w* = 1/[σ^2^(*F*_o_^2^) + (0.1056*P*)^2^ + 0.1039*P*] where *P* = (*F*_o_^2^ + 2*F*_c_^2^)/3
4470 reflections	(Δ/σ)_max_ = 0.001
213 parameters	Δρ_max_ = 0.31 e Å^−^^3^
0 restraints	Δρ_min_ = −0.23 e Å^−^^3^

### Special details

**Table:**

Geometry. All esds (except the esd in the dihedral angle between two l.s. planes) are estimated using the full covariance matrix. The cell esds are taken into account individually in the estimation of esds in distances, angles and torsion angles; correlations between esds in cell parameters are only used when they are defined by crystal symmetry. An approximate (isotropic) treatment of cell esds is used for estimating esds involving l.s. planes.
Refinement. Refinement of F^2^ against ALL reflections. The weighted R-factor wR and goodness of fit S are based on F^2^, conventional R-factors R are based on F, with F set to zero for negative F^2^. The threshold expression of F^2^ > 2sigma(F^2^) is used only for calculating R-factors(gt) etc. and is not relevant to the choice of reflections for refinement. R-factors based on F^2^ are statistically about twice as large as those based on F, and R- factors based on ALL data will be even larger.

### Fractional atomic coordinates and isotropic or equivalent isotropic displacement parameters (Å^2^)

**Table:**

	*x*	*y*	*z*	*U*_iso_*/*U*_eq_	
O1	0.70039 (15)	1.08819 (19)	0.76638 (14)	0.0819 (4)	
O2	0.66111 (13)	0.92725 (15)	0.66648 (11)	0.0630 (3)	
O3	0.28465 (12)	1.36380 (12)	1.04716 (9)	0.0495 (2)	
O4	0.04276 (15)	1.30975 (15)	1.09224 (9)	0.0602 (3)	
N1	0.39536 (14)	0.82823 (14)	0.71627 (10)	0.0439 (3)	
N2	0.16593 (13)	0.56169 (13)	0.55424 (9)	0.0411 (2)	
N3	0.32584 (19)	0.38579 (17)	0.43667 (11)	0.0598 (3)	
N4	0.61326 (13)	1.01531 (14)	0.74276 (10)	0.0457 (3)	
C1	0.1987 (2)	0.4015 (2)	0.61436 (12)	0.0579 (4)	
H1A	0.1613	0.3702	0.6912	0.069*	
C2	0.2965 (2)	0.29595 (19)	0.54111 (13)	0.0586 (4)	
H2A	0.3376	0.1779	0.5601	0.070*	
C3	0.2444 (2)	0.5452 (2)	0.44782 (13)	0.0605 (4)	
H3A	0.2415	0.6368	0.3883	0.073*	
C4	0.06090 (17)	0.72024 (18)	0.59566 (13)	0.0511 (3)	
H4A	0.0024	0.6934	0.6702	0.061*	
H4B	−0.0249	0.7878	0.5406	0.061*	
C5	0.16440 (18)	0.82458 (16)	0.60979 (12)	0.0487 (3)	
H5A	0.0879	0.9313	0.6318	0.058*	
H5B	0.2240	0.8505	0.5355	0.058*	
C6	0.29333 (16)	0.73156 (15)	0.70181 (11)	0.0423 (3)	
H6A	0.2332	0.7064	0.7760	0.051*	
H6B	0.3686	0.6242	0.6801	0.051*	
C7	0.34741 (14)	0.94586 (14)	0.78555 (10)	0.0364 (2)	
C8	0.18281 (16)	0.98950 (16)	0.84576 (11)	0.0432 (3)	
H8A	0.1076	0.9408	0.8327	0.052*	
C9	0.13241 (16)	1.10113 (16)	0.92231 (11)	0.0435 (3)	
H9A	0.0238	1.1261	0.9598	0.052*	
C10	0.23935 (15)	1.17874 (14)	0.94578 (10)	0.0383 (2)	
C11	0.39749 (15)	1.14588 (14)	0.88549 (10)	0.0379 (2)	
H11A	0.4695	1.1985	0.8982	0.045*	
C12	0.44984 (14)	1.03452 (14)	0.80583 (10)	0.0361 (2)	
C13	0.17697 (17)	1.28977 (15)	1.03544 (10)	0.0417 (3)	
C14	0.22780 (19)	1.47706 (18)	1.13211 (12)	0.0512 (3)	
H14A	0.2402	1.4115	1.2107	0.061*	
H14B	0.1087	1.5475	1.1245	0.061*	
C15	0.3332 (3)	1.5847 (2)	1.10987 (17)	0.0705 (5)	
H15A	0.2972	1.6612	1.1648	0.106*	
H15B	0.3203	1.6488	1.0319	0.106*	
H15C	0.4506	1.5140	1.1185	0.106*	
H1N1	0.495 (2)	0.808 (2)	0.6809 (15)	0.058 (5)*	

### Atomic displacement parameters (Å^2^)

**Table:**

	*U*^11^	*U*^22^	*U*^33^	*U*^12^	*U*^13^	*U*^23^
O1	0.0597 (7)	0.1035 (10)	0.1167 (11)	−0.0523 (7)	0.0326 (7)	−0.0724 (9)
O2	0.0512 (5)	0.0719 (7)	0.0792 (7)	−0.0281 (5)	0.0258 (5)	−0.0497 (6)
O3	0.0513 (5)	0.0525 (5)	0.0546 (5)	−0.0209 (4)	0.0062 (4)	−0.0319 (4)
O4	0.0660 (6)	0.0713 (7)	0.0563 (6)	−0.0357 (5)	0.0245 (5)	−0.0365 (5)
N1	0.0431 (5)	0.0441 (5)	0.0525 (6)	−0.0187 (4)	0.0085 (4)	−0.0272 (4)
N2	0.0406 (5)	0.0477 (5)	0.0404 (5)	−0.0169 (4)	0.0025 (4)	−0.0202 (4)
N3	0.0698 (8)	0.0594 (7)	0.0533 (7)	−0.0226 (6)	0.0142 (6)	−0.0296 (6)
N4	0.0406 (5)	0.0446 (5)	0.0577 (6)	−0.0183 (4)	0.0090 (4)	−0.0235 (5)
C1	0.0748 (9)	0.0574 (8)	0.0400 (6)	−0.0233 (7)	0.0052 (6)	−0.0120 (6)
C2	0.0720 (9)	0.0480 (7)	0.0528 (8)	−0.0128 (6)	−0.0063 (7)	−0.0170 (6)
C3	0.0832 (10)	0.0545 (8)	0.0434 (7)	−0.0261 (7)	0.0163 (7)	−0.0186 (6)
C4	0.0396 (6)	0.0556 (7)	0.0600 (8)	−0.0093 (5)	−0.0002 (5)	−0.0302 (6)
C5	0.0544 (7)	0.0413 (6)	0.0514 (7)	−0.0126 (5)	−0.0025 (5)	−0.0194 (5)
C6	0.0470 (6)	0.0380 (5)	0.0482 (6)	−0.0172 (5)	0.0019 (5)	−0.0203 (5)
C7	0.0416 (5)	0.0336 (5)	0.0368 (5)	−0.0146 (4)	0.0032 (4)	−0.0133 (4)
C8	0.0458 (6)	0.0454 (6)	0.0478 (6)	−0.0245 (5)	0.0110 (5)	−0.0215 (5)
C9	0.0470 (6)	0.0436 (6)	0.0454 (6)	−0.0221 (5)	0.0132 (5)	−0.0193 (5)
C10	0.0463 (6)	0.0359 (5)	0.0357 (5)	−0.0165 (4)	0.0053 (4)	−0.0144 (4)
C11	0.0416 (5)	0.0356 (5)	0.0407 (5)	−0.0157 (4)	0.0012 (4)	−0.0143 (4)
C12	0.0373 (5)	0.0344 (5)	0.0386 (5)	−0.0136 (4)	0.0043 (4)	−0.0136 (4)
C13	0.0500 (6)	0.0406 (6)	0.0379 (5)	−0.0179 (5)	0.0047 (4)	−0.0159 (4)
C14	0.0616 (8)	0.0506 (7)	0.0481 (7)	−0.0195 (6)	0.0019 (6)	−0.0275 (5)
C15	0.0860 (12)	0.0714 (10)	0.0742 (10)	−0.0428 (9)	0.0087 (9)	−0.0364 (8)

### Geometric parameters (Å, °)

**Table:**

O1—N4	1.2251 (15)	C5—C6	1.5217 (19)
O2—N4	1.2261 (14)	C5—H5A	0.9700
O3—C13	1.3313 (15)	C5—H5B	0.9700
O3—C14	1.4533 (14)	C6—H6A	0.9700
O4—C13	1.2067 (16)	C6—H6B	0.9700
N1—C7	1.3449 (13)	C7—C8	1.4249 (16)
N1—C6	1.4551 (15)	C7—C12	1.4257 (15)
N1—H1N1	0.856 (19)	C8—C9	1.3686 (15)
N2—C3	1.3395 (16)	C8—H8A	0.9300
N2—C1	1.3518 (19)	C9—C10	1.3978 (17)
N2—C4	1.4657 (15)	C9—H9A	0.9300
N3—C3	1.3120 (19)	C10—C11	1.3826 (16)
N3—C2	1.346 (2)	C10—C13	1.4840 (15)
N4—C12	1.4440 (15)	C11—C12	1.3944 (14)
C1—C2	1.351 (2)	C11—H11A	0.9300
C1—H1A	0.9300	C14—C15	1.476 (2)
C2—H2A	0.9300	C14—H14A	0.9700
C3—H3A	0.9300	C14—H14B	0.9700
C4—C5	1.5186 (19)	C15—H15A	0.9600
C4—H4A	0.9700	C15—H15B	0.9600
C4—H4B	0.9700	C15—H15C	0.9600
			
C13—O3—C14	115.30 (10)	C5—C6—H6B	108.9
C7—N1—C6	124.49 (10)	H6A—C6—H6B	107.8
C7—N1—H1N1	116.5 (12)	N1—C7—C8	119.86 (10)
C6—N1—H1N1	119.0 (12)	N1—C7—C12	125.14 (10)
C3—N2—C1	105.75 (12)	C8—C7—C12	115.00 (9)
C3—N2—C4	127.17 (12)	C9—C8—C7	121.81 (11)
C1—N2—C4	127.05 (11)	C9—C8—H8A	119.1
C3—N3—C2	104.48 (12)	C7—C8—H8A	119.1
O1—N4—O2	121.58 (11)	C8—C9—C10	121.93 (11)
O1—N4—C12	119.17 (10)	C8—C9—H9A	119.0
O2—N4—C12	119.25 (10)	C10—C9—H9A	119.0
C2—C1—N2	106.62 (13)	C11—C10—C9	118.24 (10)
C2—C1—H1A	126.7	C11—C10—C13	123.90 (11)
N2—C1—H1A	126.7	C9—C10—C13	117.86 (10)
N3—C2—C1	110.43 (13)	C10—C11—C12	120.55 (11)
N3—C2—H2A	124.8	C10—C11—H11A	119.7
C1—C2—H2A	124.8	C12—C11—H11A	119.7
N3—C3—N2	112.72 (13)	C11—C12—C7	122.29 (10)
N3—C3—H3A	123.6	C11—C12—N4	116.41 (10)
N2—C3—H3A	123.6	C7—C12—N4	121.29 (9)
N2—C4—C5	112.73 (10)	O4—C13—O3	123.64 (11)
N2—C4—H4A	109.0	O4—C13—C10	123.26 (11)
C5—C4—H4A	109.0	O3—C13—C10	113.10 (10)
N2—C4—H4B	109.0	O3—C14—C15	108.04 (12)
C5—C4—H4B	109.0	O3—C14—H14A	110.1
H4A—C4—H4B	107.8	C15—C14—H14A	110.1
C4—C5—C6	112.09 (11)	O3—C14—H14B	110.1
C4—C5—H5A	109.2	C15—C14—H14B	110.1
C6—C5—H5A	109.2	H14A—C14—H14B	108.4
C4—C5—H5B	109.2	C14—C15—H15A	109.5
C6—C5—H5B	109.2	C14—C15—H15B	109.5
H5A—C5—H5B	107.9	H15A—C15—H15B	109.5
N1—C6—C5	113.19 (11)	C14—C15—H15C	109.5
N1—C6—H6A	108.9	H15A—C15—H15C	109.5
C5—C6—H6A	108.9	H15B—C15—H15C	109.5
N1—C6—H6B	108.9		
			
C3—N2—C1—C2	0.29 (18)	C9—C10—C11—C12	−1.70 (18)
C4—N2—C1—C2	178.23 (13)	C13—C10—C11—C12	177.37 (10)
C3—N3—C2—C1	−0.4 (2)	C10—C11—C12—C7	−2.12 (18)
N2—C1—C2—N3	0.0 (2)	C10—C11—C12—N4	176.86 (11)
C2—N3—C3—N2	0.6 (2)	N1—C7—C12—C11	−175.11 (11)
C1—N2—C3—N3	−0.5 (2)	C8—C7—C12—C11	4.66 (17)
C4—N2—C3—N3	−178.48 (13)	N1—C7—C12—N4	5.96 (19)
C3—N2—C4—C5	−70.00 (19)	C8—C7—C12—N4	−174.27 (11)
C1—N2—C4—C5	112.49 (16)	O1—N4—C12—C11	4.07 (19)
N2—C4—C5—C6	−62.97 (15)	O2—N4—C12—C11	−175.55 (12)
C7—N1—C6—C5	85.02 (15)	O1—N4—C12—C7	−176.93 (13)
C4—C5—C6—N1	179.45 (10)	O2—N4—C12—C7	3.44 (19)
C6—N1—C7—C8	−3.56 (19)	C14—O3—C13—O4	−1.8 (2)
C6—N1—C7—C12	176.19 (11)	C14—O3—C13—C10	178.66 (10)
N1—C7—C8—C9	176.17 (12)	C11—C10—C13—O4	−175.61 (13)
C12—C7—C8—C9	−3.61 (18)	C9—C10—C13—O4	3.5 (2)
C7—C8—C9—C10	0.0 (2)	C11—C10—C13—O3	3.97 (18)
C8—C9—C10—C11	2.76 (19)	C9—C10—C13—O3	−176.96 (11)
C8—C9—C10—C13	−176.37 (12)	C13—O3—C14—C15	−163.51 (13)

### Hydrogen-bond geometry (Å, °)

**Table:**

*Cg*1 is the centroid of the C7–C12 benzene ring.

**Table:**

*D*—H···*A*	*D*—H	H···*A*	*D*···*A*	*D*—H···*A*
N1—H1N1···O2	0.859 (18)	2.004 (18)	2.6464 (18)	130.9 (15)
N1—H1N1···N3^i^	0.859 (18)	2.345 (17)	3.0281 (18)	136.7 (15)
C15—H15A···O1^ii^	0.96	2.47	3.346 (2)	151
C1—H1A···Cg1^iii^	0.93	2.90	3.5962 (16)	132

Symmetry codes: (i) −*x*+1, −*y*+1, −*z*+1; (ii) −*x*+1, −*y*+3, −*z*+2; (iii) *x*, *y*−1, *z*.
